# Causes and clinical presentation of stroke in children in Cameroon

**DOI:** 10.4314/gmj.v59i1.2

**Published:** 2025-03

**Authors:** Daniel A Kago-Tague, Fabricia N Guimeya, Joseph Kamtchum-Tatuene, Dominique Enyama, Euranie J Kouam, Hubert D Mbassi, Seraphin Nguefack

**Affiliations:** 1 Faculty of Medicine and Biomedical Sciences, University of Yaoundé I, Yaoundé, Cameroon; 2 Wolfson Centre for Prevention of Stroke and Dementia, Nuffield Department of Clinical Neurosciences, University of Oxford, Oxford, United Kingdom; 3 Faculty of Medicine and Pharmaceuticals Sciences, University of Dschang, Dschang, Cameroon

**Keywords:** Stroke, Children, Cameroon

## Abstract

**Objective:**

The aim was to determine the aetiological factors and clinical and paraclinical aspects of stroke in children in Cameroon.

**Design:**

retrospective study of the records

**Setting:**

At two university hospitals in the city of Yaoundé (Yaoundé Gynaeco-Obstetric and Paediatric Hospital and the Chantal Biya Foundation Mother and Child Centre)

**Participants:**

47 children with stroke for seven and half years

**Interventions:**

Data were collected from medical records. The variables studied included clinical and paraclinical data.

**Main outcome measures:**

Key variables were summarised in the form of mean ± standard deviation, frequencies and percentages

**Results:**

The mean age was 6.5±2.8 years. The Male Female sex ratio was 1.8:1. The average consultation time was 31.8 hours. Hemiplegia/hemiparesis (95.7%) was the main clinical manifestation, associated with signs such as convulsions (27.7%), fever (46.8%) and pallor (27.7%). Ischaemic and haemorrhagic stroke accounted for 41 cases (87.2%) and 6 cases (12.8%), respectively. The aetiological factors for ischaemic stroke were sickle cell disease (72.3%), sepsis (4.2%), protein S deficiency (2.1%) and dilated cardiomyopathy with mitral insufficiency (2.1%). The aetiology was not found in 3 patients (6.4%) with ischaemic stroke. Apart from sickle cell disease (66.6%), the aetiological factors for haemorrhagic stroke were idiopathic thrombocytopenic purpura (16.7%) and haemophilia B (16.7%). Ischaemia mainly involved the middle cerebral artery (86.1%). Haemorrhagic attacks were mainly supratentorial.

**Conclusion:**

In urban Cameroon, strokes frequently occur around the age of 6, with a predominance of ischaemic strokes resulting in motor deficits. Sickle cell disease is the most common cause.

**Funding:**

None declared

## Introduction

Stroke is a focal neurological deficit that persists for more than 24 hours and is caused by stenosis, occlusion or rupture of cerebral blood vessels.^1^-^3^ It may be arterial, venous or due to occlusion of the venous sinuses. The incidence of stroke in children varies from 2.5 to 13 per 100,000 per year.^4^ Ten to 25% of children who suffer a stroke die, 25% have a recurrence, and around 66% suffer a neurological deficit that persists beyond the acute phase or has epilepsy, behavioural problems or learning difficulties.^4^,^5^

The risk factors for stroke in children are numerous and differ greatly from those in adults.^6^ The coexistence of several stroke risk factors in a patient increases the risk of stroke recurrence and mortality.^7^ Heart diseases and haemoglobinopathies are the most common causes of ischaemic infarction, while various congenital blood vessel anomalies and haemostasis disorders are often found in children with intra-parenchymal haemorrhage.^8^-^10^

Identifying these factors can help prevent recurrence and reduce morbidity. Hence, this study aimed to determine the aetiological factors and clinical and paraclinical aspects of stroke in children in Cameroon.

## Methods

This was a 7-and-a-half-year retrospective study from 1 January 2015 to 31 July 2022. The study took place in two university hospitals in Yaoundé (Yaoundé Gynaeco-Obstetric and Paediatric Hospital and Chantal Biya Foundation Mother and Child Centre). The Institutional Ethics Committee approved this study for Human Research of the Faculty of Medicine and Biomedical Sciences of the University of Yaoundé I (N° 246 /UY1 /FMSB /VDRC /DAARSR /CSD), and we received research authorisations from the administrative managers of the various study sites. Children aged between 1 month and 18 years whose diagnosis of stroke had been confirmed by imaging were included.

Data were collected from medical records. Patients with incomplete records were excluded. The variables studied included clinical data (age, sex, history, mode of onset, time to admission, motor deficit and associated signs). The paraclinical data sought were cerebral imaging (type of lesion, vascular topography) and the results of complementary examinations for the aetiological investigation of the stroke. Continuous variables were described by the parameters of central tendency (mean) or dispersion (standard deviation, maximum, minimum). Categorical variables were described in terms of frequencies and percentages. Statistical analysis was performed using SPSS version 28 software.

## Results

During the study period, 73225 children were admitted to the two hospitals, including 47 cases of paediatric stroke, representing a prevalence of 0.06% with 30 boys and 17 girls (sex ratio 1.8:1). The mean age of the patients was 6.5 ± 2.8 years, with extremes of 11 months and 14 years. The 5 to 10 age group was the most represented with 30 patients (63.9%), while 23.4% (11/47) were between one month and 5 years old and 12.8% (6/47) were over 10 years old.

A child could have several reasons for hospital admission. The main reasons for consultation were motor deficits (51%) and convulsions (27.7%). The average consultation time at one of our two sites after the onset of signs and symptoms was 31.8 hours, with extremes ranging from 1 hour to 168 hours. The medical histories reported were sickle cell disease in 31 cases (65.9%), varicella in 2 children (4.3%), haemophilia B (1 case or 2.1%) and idiopathic thrombocytopenic purpura (1 case or 2.1%). The clinical neurological picture on admission was dominated by a motor deficit (95.7%), facial paralysis (59.6%), aphasia (34%), convulsions (27.7%) and coma (27.7%). This picture was associated with fever (46.8%) and mucocutaneous pallor (27.7%) (Table 1).

**Table 1:** Distribution of stroke patients according to clinical signs

Clinical signs	Frequency (n = 47) n(%)
Neurological signs	
Hemiplegia/Hemiparesis	45 (95.7)
Facial palsy	28 (59.6)
Aphasia	16 (34.0)
Convulsions	13 (27.7)
Coma	13 (27.7)
Meningeal signs	4 (8.5)
Intracranial hypertension	3 (6.4)
Extra-neurological signs	
Fever	22 (46.8)
Pallor	13 (27.7)
Arterial hypertension	6 (12.8)
ENT infection	1 (2.1)
Haemorrhagic syndrome	1 (2.1)
Heart murmur	1 (2.1)

Note: Brain imaging was without abnormality in 5 patients.

Cerebral computed tomography was performed in 44 patients (93.6%), while cerebral magnetic resonance imaging (MRI) was performed in 3 patients (6.4%). Ischaemic and haemorrhagic strokes accounted for 41 cases (87.2%) and six cases (12.8%), respectively. Of the patients with ischaemic stroke, 5 had a CT scan described as normal. The territory of the middle cerebral artery was the most affected (86.1%) by ischaemia (Table 2). Haemorrhagic transformation of the ischaemia was present in 3 (8.3%) patients.

**Table 2:** Arterial territories of cerebral ischaemia and topography of cerebral haemorrhage

Arterial territories of cerebral ischaemia	Frequency n(%) (n=36)	Topography of cerebral haemorrhage	Frequency n(%) (n=6)
Middle cerebral artery	31 (86.1%)	Subarachnoid	1 (16.7%)
Anterior cerebral artery	2 (5.5%)	Frontal	1 (16.7%)
Anterior and middle cerebral artery	2 (5.5%)	Parietal	1 (16.7%)
Cerebellar artery	1 (2.8%)	Temporal	1 (16.7%)
		Temporo-parietal	2 (33.3%)

The aetiological factor most frequently identified was sickle cell disease, present in 38 patients (80.9%). Stroke was the mode of detection of sickle cell disease in 7 patients (7/38, 18.4%). Recurrent stroke was found in 9 patients with sickle cell disease (19.1%). The mean age at onset of stroke in patients with sickle cell disease was 81 months (6 years 9 months), with extremes of 34 months (2 years 10 months) and 14 years.

Apart from sickle cell disease (66.6%), the other aetiological factors for haemorrhagic stroke were idiopathic thrombocytopenic purpura and haemophilia B, while protein S deficiency, dilated cardiomyopathy with mitral insufficiency and sepsis were the other aetiologies for ischaemic stroke. No aetiology was found in 3 patients (7.3%) with ischaemic stroke (Table 3).

**Table 3:** Aetiologies of stroke

Causes	Ischaemic stroke (n=41)	Haemorrhagic stroke (n=6)	Total n(%) (n=47)
Sickle cell disease	34	4	38 (80.9%)
Protein S deficiency	1	0	1 (2.1%)
Thrombocytopaenia	0	1	1 (2.1%)
Haemophilia B	0	1	1 (2.1%)
Cardiomyopathy	1	0	1 (2.1%)
Sepsis	2		2 (4.3%)
Aetiology not found	3		3 (6.4%)

None of the patients required thrombolysis. Therapeutic measures were used on a case-by-case basis, in particular, hydration and transfusion of packed red blood cells in the 13 sickle cell patients. No patient with sickle cell disease received an exchange transfusion. Two deaths occurred, both from haemorrhagic stroke.

## Discussion

In urban Cameroon, strokes occur in older children (6 years), are predominantly ischaemic and present with motor deficits. Sickle cell disease is the most common cause. The mean age was 6.5 years, which is consistent with the literature, where it varies between four and 13 years.^9^,^11^,^12^ This age also corresponds to the age of onset of stroke in patients with sickle cell disease. As described by other authors, we found a male predominance.^13^,^14^

As reported by Tohodjede et al. 13, we noted a delay in diagnosis due to the delay in imaging, with a mortality rate of 5%, rising to 15% in the event of recurrence and long-term sequelae.^5^,^15^ When a positive diagnosis of cerebral arterial infarction is made within 4 hours and 30 minutes of the onset of signs, thrombolytic treatment may be considered on a case-by-case basis.^5^

In our patients, hemiplegia was the most common mode of stroke onset, accompanied by language impairment, indicating involvement of the dominant hemisphere. These results are comparable to those found by other authors.^11^,^12^,^14^ This could be explained by the involvement of the middle cerebrum, which was predominant in our study, resulting in a lesion of the pyramidal bundle.^16^ Signs associated with motor deficit in this study were convulsions, coma, fever and pallor. Thiam et al.^9^ in Senegal in 2022 found convulsive seizures, hyperthermia and consciousness disorders. The occurrence of convulsions, most often with a focal component, is a paediatric specificity observed in 30% of children, particularly the youngest.^5^ In adults, the incidence of convulsions associated with stroke is lower (generally 10%).^17^ Coma may reveal forms that are initially very severe.^5^

As described in the literature, ischaemic stroke was more common.^4^,^7^,^10^ The main aetiological factor in our series was sickle cell disease (80.9%), the ischaemic complications of which result from vascular occlusions occurring at the time of sickle cell crises.^18^ Our results are identical to those of other authors in Africa.^8^,^13^,^18^,^19^ Stroke is one of the most serious and frequent neurological complications of sickle cell disease, with a prevalence of 4.01%.^3^,^20^,^21^ Stroke was the route of diagnosis in 7 cases of sickle cell disease in our series. This could be explained by the absence of systematic screening for sickle cell disease at birth in Cameroon. Recurrence was observed in one child out of 5. Ohene et al.^3^ described a recurrence rate of 14% within an average of 3 months after the initial stroke. Increased preventive measures such as neonatal screening, regular monitoring by transcranial Doppler ultrasound and improved access to hydroxycarbamide would reduce the incidence of stroke in children with sickle cell disease.^20^

The aetiology was not found in 3 patients (7.3%) with ischaemic stroke. In addition to the fact that CT scans do not always allow early diagnosis of cerebral arterial infarction, they do not identify the most frequent differential diagnoses (migraine with aura, post-critical deficit), which require urgent appropriate neuropaediatric management.^5^,^22^ Unlike other African authors, we found one case of heart disease. Heart disease with right-left shunts is a major cause of cerebrovascular accidents because it is responsible for polycythaemia, which can favour thrombosis.^5^,^7^,^11^,^18^

A febrile context was noted in almost half the patients, suggesting an infection, but the aetiological investigation was usually inconclusive due to the inaccessibility of certain biological analyses. Stroke due to infectious arteritis accounted for 9% of the causes of ischaemic stroke in the study by Mbaye et al.^11^ Viral infections, particularly upper respiratory infections, and varicella, as found in our study, are described as the most frequent causes of transient focal arteriopathy.^5^ These infections are thought to lead to a state of hypercoagulability due to a transient drop in the free fraction of protein S, particularly in the case of viral infections.^23^,^24^

Protein S deficiency, which plays a cofactor role in coagulation by inhibiting factors V and VIII, was found in one patient. It increases the risk of thrombogenesis. It is a rarely implicated aetiology, as noted by a multicentre study in several countries, which reported five cases of protein S deficiency.^4^

Apart from sickle cell disease, the two other aetiologies of haemorrhagic stroke that we found were idiopathic thrombocytopenic purpura and haemophilia B. However, according to other authors, the main causes of haemorrhagic stroke are coagulopathies, arteriovenous malformations and brain tumours. Cerebral malformations and tumours could have been identified by MRI, which is the reference diagnostic imaging for stroke in children but is rarely used in our context.^5^,^22^ Xie et al.^25^ in China reported more cases of haemorrhagic stroke (64.5%) than ischaemic stroke (35.5%). In their series, the main aetiologies were coagulopathies (59.6%), vitamin K deficiency, haemophilia and thrombocytopenia, followed by vascular malformations (8.26%).^25^ We found three cases of haemorrhagic transformation in the territories of the cerebral artery. Haemorrhagic transformation may occur spontaneously in infarcted tissue or be a complication of thrombolysis.^26^

## Conclusion

In urban Cameroon, strokes in children were found to occur around the age of six, with a predominance of ischaemic strokes. They manifest as a sudden onset of unilateral motor deficit (hemiplegia/hemiparesis), which may be associated with acute speech impairment, convulsions and fever. Sickle cell disease is the most common cause. Systematic neonatal screening for sickle cell disease and proper management of known sickle cell patients would reduce the frequency of childhood stroke in our context.
